# A phase I study of farletuzumab, a humanized anti-folate receptor α monoclonal antibody, in patients with solid tumors

**DOI:** 10.1007/s10637-014-0180-8

**Published:** 2014-11-09

**Authors:** Yasutsuna Sasaki, Keisuke Miwa, Keishi Yamashita, Yu Sunakawa, Ken Shimada, Hiroo Ishida, Kosei Hasegawa, Keiichi Fujiwara, Makoto Kodaira, Yasuhiro Fujiwara, Masayuki Namiki, Minami Matsuda, Yutaka Takeuchi, Noriyuki Katsumata

**Affiliations:** 1 Department of Medical Oncology, Saitama International Medical Center-Comprehensive Cancer Center, Saitama Medical University, Saitama, Japan; 2 Department of Gynecologic Oncology, Saitama International Medical Center-Comprehensive Cancer Center, Saitama Medical University, Saitama, Japan; 3 Department of Breast and Medical Oncology, National Cancer Center Hospital, Tokyo, Japan; 4 Eisai Co., Ltd., Tokyo, Japan; 5 Present Address: Division of Medical Oncology, Department of Medicine, Showa University School of Medicine, Tokyo, Japan; 6 Present Address: Multidisciplinary Treatment Cancer Center, Kurume University Hospital, Fukuoka, Japan; 7 Present Address: Kawagoe Gastroenterical Hospital, Saitama, Japan; 8 Present Address: Division of Medical Oncology, Department of Medicine, Showa University Northern Yokohama Hospital, Yokohama, Japan; 9 Present Address: Division of Medical Oncology, Department of Medicine, Showa University Koto-Toyosu Hospital, Tokyo, Japan; 10 Present Address: Department of Medical Oncology, Nippon Medical School Musashikosugi Hospital, Kawasaki, Japan

**Keywords:** Farletuzumab, Folate receptor α, Monoclonal antibody, Pharmacokinetics, Phase I study

## Abstract

Farletuzumab is a humanized monoclonal antibody against folate receptor α (FRA). The purpose of the study is to assess safety and tolerability, the pharmacokinetic (PK) profile, and preliminary antitumor effect. Patients with ovarian cancer (OC) or FRA-expressing solid tumors who are resistant to standard treatments were eligible for the study. After single-dose administration for PK assessment, farletuzumab was administered by intravenous injection, repeating every week until disease progression. Dose-limiting toxicities (DLTs) were defined as grade 4 hematological and grade 3/4 nonhematological toxicities. Dose escalation was planned in 4 cohorts (50, 100, 200, and 400 mg/m^2^). Fourteen patients with OC and two patients with gastric cancer (GC) received farletuzumab infusion. Neither DLTs nor grade 3/4 toxicities were reported in all cohorts. Major adverse events, including grade 1/2 infusion related reaction (15 patients, 93.8 %), headache (seven patients, 43.8 %), and nausea and decreased appetite (five patients each, 31.3 %), were observed and medically managed. AUC and C_max_ increased dose-dependently and linear PK profiles were observed. No tumor shrinkage was recorded, but long-term disease stabilization for 25 and 20 months was observed in one patient with clear cell OC (100 mg/m^2^) and one patient with GC (400 mg/m^2^), respectively. No cumulative toxicity occurred in any patient. Farletuzumab was well tolerated in Japanese patients with a similar PK profile as compared with the US population. Long-term disease stabilization was observed in a subpopulation of clear cell OC and GC; both of them were resistant and progressive after standard chemotherapies (ClinicalTrials.gov Identifier: NCT01049061).

## Introduction

Cancer is the most common and life-threatening disease worldwide whose incidence continues to increase. In Japan, one half of the population has a chance to be diagnosed with cancer in their life time and one third of these Japanese patients lose their lives by cancer, especially lung, gastric, and colorectal cancers [1, 2]. In addition to surgery or radiotherapy, new medical treatment options including chemotherapy and targeting therapies that are necessary to improve therapeutic outcomes especially in patients with metastatic or recurrent cancer.

Folate plays important roles for DNA synthesis and repair in proliferating cancer cells compared to normal cells [3]. Folate receptor α (FRA) is a protein with high affinity for binding and transporting physiologic levels of folate into cells [4]. High expression of FRA are observed in specific malignant tumors including ovarian cancer, nasopharyngeal epidermoid carcinoma, cervical carcinoma, uterine carcinoma, primary renal cell carcinoma and metastatic pancreatic carcinoma [3]. Protein expression in FRA-positive tumor, nonmucinous epithelial ovarian carcinoma, is associated with tumor progression, and also with platinum-therapy resistance, and poor prognosis [3].

Farletuzumab (MORAb-003; Morphotek, Inc.) is a humanized monoclonal antibody immunoreactive with human FRA [5]. Expression of FRA is identified especially in over 90 % of serous ovarian cancers (OC) [6–8]. Farletuzumab mediates tumor cytotoxicity via antibody-dependent cell cytotoxicity (ADCC) and complement-dependent cytotoxicity (CDC) of an FRA-expressing human OC cell line in vitro [5, 9]. Farletuzumab reduces tumor growth in FRA-expressing OC cells in vivo in a xenograft model [5, 9]. The first disease-oriented phase I trial of farletuzumab for OC patients, which was conducted by Konner, reported a promising disease-stabilizing effect and reduction of serum CA125 in patients with heavily pretreated OC [10]. A subsequent phase II study suggests that farletuzumab in combination with carboplatin/taxane, followed by single-agent farletuzumab maintenance, enhance the response rate and duration of response in platinum-sensitive ovarian cancer patients with first relapse after a remission of 6 to 18 months [11]. However, little information is available as to the anticancer effect of farletuzumab for FRA-expressing non-OC, and no information is reported regarding the relationship between expression level of FRA and antitumor effects [10, 11].

We conducted a phase I trial of farletuzumab in Japanese patients with solid tumors and analyzed the relationship between FRA expression level and clinical outcome. This is the first clinical study of farletuzumab that targets not only OC but also FRA-expressing non-OC patients.

## Materials and methods

### Trial objectives

This was a single arm, open-label, dose escalation phase I trial to determine maximum tolerated dose (MTD) by evaluating dose-limiting toxicities (DLTs) as the primary endpoint. Secondary endpoints included investigation of safety and tolerability, estimation of recommended dose (RD) for the next studies, evaluation of antitumor effects, investigation of the pharmacokinetic (PK) profiles of farletuzumab, and detection of human anti-human antibody (HAHA). In addition, a comparison of PK profiles of farletuzumab between Japanese and non-Japanese populations was performed. Progression-free survival (PFS) by FRA expression level was also assessed in subjects whose tumor tissue samples were available. This study was conducted at Saitama Medical University International Medical Center (Hidaka, Saitama) and National Cancer Center Hospital (Tokyo) in Japan.

### Patient selection

Patients who had histologically documented OC regardless of FRA expression were evaluable for the study. Patients with non-OC were also eligible for the study if FRA-expression was confirmed by IHC staining. Patients must have had no standard therapy or had failed prior standard therapies at the time of registration. Other inclusion criteria were as follows: Eastern Cooperative Oncology Group (ECOG) performance status (PS) of 0 or 1; age ≥20 and ≤80 years; life expectancy ≥12 weeks; and adequate major organ function (i.e., hemoglobin ≥10.0 g/dL; white blood cell count ≥3,000 /mm^3^, <12,000 /mm^3^; platelet count ≥100,000/mm^3^; total bilirubin ≤2.0 mg/dL; alanine transaminase (ALT), aspartate transaminase (AST), and alkaline phosphatase ≤2.5 times upper limit of normal (ULN) or 5 times ULN in the presence of liver metastases; serum creatinine <2.0 mg/dL).

Exclusion criteria were symptomatic metastatic brain tumors; serious and systemic infection requiring medical treatment; HIV, hepatitis C antibody, or Hepatitis B surface antigen positive, history of cardiac infarction within 6 months; uncontrolled cardiac disease; liver cirrhosis or interstitial lung disease; active synchronous cancer; history of hypersensitivity to monoclonal antibody or protein formulation; pleural effusion, ascites or pericardial effusion necessary for drainages; and pregnancy or breast feeding.

### Drug administration and dose escalation

Single intravenous doses of farletuzumab were administered in cycle 0 on day 1 to monitor the PK profile. From cycle 1 onward, farletuzumab was administered on days 1, 8, 15, and 22 once a week for 4 weeks as one cycle. Farletuzumab was continued weekly, until disease progression or DLT was observed in the patients. DLT was defined as a treatment-related adverse event (AE) that was relevant to any of the following two different criteria: (i) hematologic toxicity, which included grade 4 leucopenia or neutropenia persisting for 7 days or more, ≥ grade 3 febrile neutropenia, grade 4 thrombocytopenia, or grade 3 thrombocytopenia requiring blood transfusion; (ii) nonhematologic toxicity ≥ grade 3, excluding abnormal laboratory test results not requiring treatment, nausea, vomiting, and diarrhea.

Farletuzumab was administered by intravenous continuous infusion starting at 1–2 mg/min and advancing to 5 mg/min if no AE greater than grade 1 was observed. Premedication for allergic reaction was not allowed before the first dosing. The dose escalation strategy was determined based on a previous phase I study [10] and adapted to a conventional 3 + 3 method. The starting dose of farletuzumab was decided to be 50 mg/m^2^. Dose escalation was followed by 100, 200, and 400 mg/m^2^ doses. The maximum dose level of 400 mg/m^2^ was adjusted as in the previous phase I study. If DLT was observed in any planned cohorts within 7 weeks of initiating therapy (cycle 0 and cycle 1), up to six patients were to be entered at that dose level. Dose escalation was permitted only after all patients in the cohort had completed cycle 0 and cycle 1. Intrapatient dose escalation was not permitted.

### Safety and efficacy assessment

Patients were assessed through physical examination, vital signs, complete blood count, chemistries, urinalysis, and ECOG PS. AEs were evaluated at each study visit and graded according to Common Terminology Criteria for Adverse Events (CTCAE) version 3.0 [12]. Immune-mediated AEs including allergic reaction were classified as adverse events of interest based on the judgment by investigators and summarized as infusion related reactions in this paper. Radiological assessment for tumor response was performed at baseline, cycle 1, and every 8 weeks thereafter. Tumor response was evaluated according to Response Evaluation Criteria in Solid Tumors (RECIST) version 1.0 [13]. PFS was defined as the interval between the first administration of study drug and the first radiographical documentation of disease progression or death from any cause (whichever occurred first). The prior lower dose level below which two or more of six patients experienced DLT was determined to be the maximum tolerated dose (MTD).

### Detection of FRA expression

FRA expression was examined with IHC in patients with solid tumors except for OC. For patients with OC, IHC was performed only for those whose tissue samples were available. Paraffin-embedded samples (3 to 5 μm slices, at least 3 slices) were placed on glass slides and refrigerated. Frozen samples were transferred into polypropylene tubes to be stored at −80 °C until shipment. The collected samples (either paraffin-embedded or frozen) were shipped to the biomarker measurement laboratory (Mitsubishi Chemical Medience, Ibaraki, Japan) on the same day, as feasible. Tumor FRA expression was evaluated using the commercially available anti-FRA antibody, Novocastra Liquid Mouse Monoclonal Antibody Folate Receptor alpha (Leica Biosystems, Newcastle, UK). Pathologists performed grading for staining manually. Immunoreactive intensities were graded as no reaction (−), weak (+: stained light brown with a thin rim along the cell membrane), moderate (++: stained deep-brown with a thin rim along the cell membrane), or strong (+++: stained deep-brown with a thick rim along the cell membrane).

### Pharmacokinetic analysis

Blood sampling for PK analysis was performed for all patients in cycle 0 on day 1 at predose, 30 min after starting infusion, just after finishing infusion, 30 min, 1, 2, 4, 24, 72, 168, 336 h after infusion. In addition, blood samples at predose in cycle 1 on days 22, 29, 36, and 43 and 30 min after start of infusion, just after finishing infusion, 30 min, 1, 2, 4 h after infusion on day 43 were also collected.

Serum concentrations of farletuzumab were determined by a validated enzyme-linked immunosorbent assay. Serum farletuzumab levels from 0.313 to 2,000 μg/mL could be determined by this assay. The assay was performed by Catalent Pharma Solutions, LLC (Morrisville, NC). The following PK parameters were calculated by non-compartmental method using WinNonlin™ Professional (version 6.2.1; Pharsight Corporation Mountain View, CA): maximum observed serum concentration (C_max_), time at which the highest serum drug concentration occurred (t_max_), area under the serum concentration-time curve (AUC), terminal phase rate constant (λ_z_), terminal elimination half-life (t_1/2_), and distribution volume at terminal phase (V_z_). Pharmacokinetic data of a previously reported phase I study [10] was used as reference for comparison of PK profiles between Japanese and US patients.

### Detection of HAHA

Blood samples for HAHA analysis were collected before farletuzumab administration on day 1 and 336 h after infusion on day 15 in cycle 0, preinfusion on days 1 and 15 in cycle 1, preinfusion on day 1 in cycle 2 and later, at study discontinuation, and at 30 days after the last dose. Analysis was performed by Morphotek, Inc. (Exton, PA). HAHA responses in human serum were detected by a validated electrochemiluminescence (ECL) quasi-quantitative immuno-bridging assay. Farletuzumab was used as the capture antigen, and the biotin-conjugate by virtue of horseradish peroxidase-enzyme conjugation was used as the detection reagent in a bridging assay format. HAHA complexes were subsequently captured on streptavidin-coated microtiter plates and detected in a quasi-quantitative manner via ECL signal generation.

## Results

### Patient characteristics

**Fig. 1:**
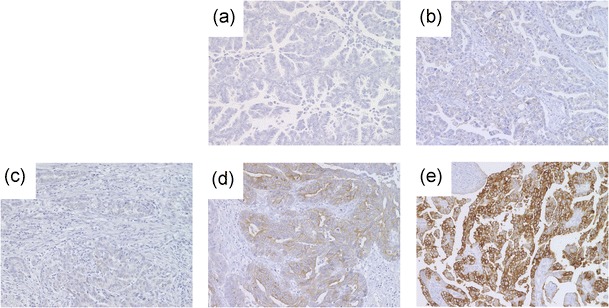
Representative images of immunohistochemical staining for folate receptor α. **a** Patient #7 (ovarian cancer; negative staining). **b** Patient #6 (ovarian cancer; + staining). **c** Patient #16 (gastric cancer; + staining). **d** Patient #15 (ovarian cancer; ++ staining). **e** Patient #1 (ovarian cancer; +++ staining)

From December 2009 to March 2011, 16 patients were enrolled in this study and received at least one intravenous infusion of farletuzumab. Patient demographics are listed in Table 1. The median age was 57 years (range, 35–72 years). Eleven patients (68.8 %) had an ECOG PS score of 0 and five (31.3 %) had a PS of 1. Fourteen female patients had OC and two male patients had gastric cancer (GC). All patients had received both surgery and more than two prior chemotherapeutic regimens. Twelve OC patients and 2 GC patents were examined for FRA expression by staining of archival tumor tissue obtained at surgery but not just before farletuzumab treatment. Eleven OC patients (91.7 %) and 2 GC (100 %) patients were FRA-positive. One patient’s OC, deemed FRA-negative, was of unclassified histology. Representative images of immunohistochemical staining for FRA are shown in Fig. 1. Each patient received farletuzumab at one of the dose levels from 50 mg/m^2^ to 400 mg/m^2^ according to the dose escalation strategy. Rapid disease progression was observed in one patient (#13) at the 400 mg/m^2^ dose level before DLT evaluation and another patient was added to replace this patient.

**Table 1:** Patient demographics

Patient #	Dose (mg/m^2^)	Sex	Age	ECOG PS	Disease	Prior regimens	FRA expression (intensity)
# 1	50	Female	72	0	OC (serous)	2	Positive (+++)
# 2	50	Female	66	1	OC (serous)	3	Positive (++)
# 3	50	Female	62	0	OC (serous)	6	Positive (+)
# 4	100	Female	41	0	OC (clear cell)	4	Positive (+)
# 5	100	Female	57	0	OC (serous)	3	Positive (+++)
# 6	100	Female	48	0	OC (clear cell)	2	Positive (+)
# 7	200	Female	35	0	OC (unclassified)	3	Negative (−)
# 8	200	Female	70	1	OC (serous)	6	NE
# 9	200	Female	46	1	OC (clear cell)	3	NE
# 10	400	Male	71	0	GC (intestinal)	2	Positive (+)
# 11	400	Female	50	0	OC (serous)	4	Positive (+++)
# 12	400	Female	52	0	OC (serous)	3	Positive (++)
# 13	400	Female	58	0	OC (unclassified)	5	Positive (++)
# 14	400	Female	48	1	OC (serous)	4	Positive (++)
# 15	400	Female	57	1	OC (endometrioid)	7	Positive (++)
# 16	400	Male	63	0	GC (diffuse)	3	Positive (+)

*ECOG PS* Eastern Cooperative Oncology Group performance status; *FRA* folate receptor α; *OC* ovarian cancer; *GC* gastric cancer; *NE* not examined

### Safety

A total of 124 treatment-related AEs (adverse reactions) were reported in the 16 patients during the study. Treatment-related AEs occurring in at least 15 % of patients are shown in Table 2. There were no DLTs in cycle 0 to cycle 1 in the all treatment cohorts. However, all patients experienced at least one adverse reaction during the study. The majority of adverse reactions in this phase I study were grade 1, and no adverse reactions of grade 3 or higher were observed. There was no clear relationship between dose of farletuzumab and frequency or severity of adverse reactions for the whole study period. No cumulative adverse reactions leading to discontinuation from study treatment were reported. The major adverse reactions were infusion related reaction (15 patients, 93.8 %), headache (7 patients, 43.8 %), and nausea and decreased appetite (5 patients each, 31.3 %). All infusion related reactions reported during the study period occurred during or after the first infusion in cycle 0. All the reactions developed within 24 h after the start of farletuzumab administration and resolved within 72 h after onset. HAHA was not detected in any samples. Although elevation of ALT and AST was observed, all occurrences were classified as grade 1 and were reversible.

**Table 2:** Treatment-related adverse events and incidence occurring in at least 15 % of patients

Treatment-related adverse events	Grade 1 *n* (%)	Grade 2 *n* (%)	Total *n* (%)
Infusion related reaction	7 (43.8)	8 (50.0)	15 (93.8)
Headache	6^a^ (37.5)	1 (6.3)	7^a^ (43.8)
Nausea	5^a^ (31.3)	0	5^a^ (31.3)
Decreased appetite	5 (31.3)	0	5 (31.3)
Alanine aminotransferase increased	4 (25.0)	0	4 (25.0)
Aspartate aminotransferase increased	4 (25.0)	0	4 (25.0)
Fatigue	4 (25.0)	0	4 (25.0)
Abdominal pain	3 (18.8)	0	3 (18.8)
Blood urine present	3 (18.8)	0	3 (18.8)

MedDRA Version 14.1

^a^One patient is also counted as having an infusion related reaction

### Pharmacokinetics

**Fig. 2:**
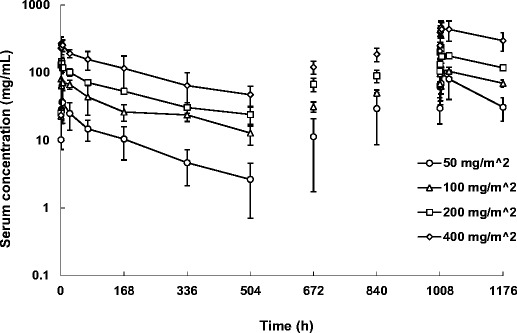
Mean serum concentrations of farletuzumab after administration up to cycle 1 (*n* = 3 in 50, 100, and 200 mg/m^2^-dose group; *n* = 7 in 400 mg/m^2^-dose group). Error bars show standard deviation

The mean serum farletuzumab concentration-time profile up to cycle 1 is shown in Fig. 2. Farletuzumab was eliminated biphasically after reaching C_max_. Pharmacokinetic parameters on cycle 0 day 1 and on cycle 1 day 22 are shown in Table 3. The mean C_max_ on cycle 0 day 1 ranged from 40.6 μg/mL to 293.4 μg/mL, and the mean AUC_(0-t)_ on cycle 0 day 1 ranged from 4772.7 to 48614.0 μg•h/mL. C_max_ and AUC_(0-t)_ increased almost dose dependently. The mean t_1/2_, CL, and V_ss_ on cycle 0 day 1 were 152.6 to 277.7 h, 5.30 to 10.54 mL/h/m^2^, and 1.87 to 2.29 L/m^2^, respectively. These results revealed that farletuzumab was gradually eliminated with a low clearance and a low distribution volume. Meanwhile, the mean C_max_ on cycle 1 day 22 ranged from 97.4 to 535.5 μg/mL, and the mean AUC_(0-t)_ and t_1/2_ values on cycle 1 day 22 ranged from 10134.5 to 72055.3 μg•h/mL and 213.4 to 276.1 h, respectively.

**Table 3:** Pharmacokinetic parameters after farletuzumab infusion on day 1 of cycle 0 and day 22 of cycle 1

Parameter	Dose of farletuzumab			
	50 mg/m^2^	100 mg/m^2^	200 mg/m^2^	400 mg/m^2^
Day 1 of cycle 0	*n* = 3	*n* = 3	*n* = 3	*n* = 7
C_max_ (μg/mL)	40.6 ± 12.3	81.6 ± 14.4	154.6 ± 10.6	293.4 ± 60.8
T_max_ (h)	2.78 ± 1.49	2.63 ± 2.37	3.39 ± 1.50	4.89 ± 1.61
AUC_(0-t)_ (μg•h/mL)	4772.7 ± 2115.9	14945.5 ± 2630.4	24060.3 ± 1788.8	48614.0 ± 15012.3
AUC_(0-inf)_ (μg•h/mL)	5420.1 ± 2648.1	20000.1 ± 5471.8	34286.8 ± 7902.0	69704.9 ± 17583.6^a^
t_1/2_ (h)	152.6 ± 37.3	255.1 ± 94.5	277.7 ± 83.5	229.7 ± 82.0^a^
CL (mL/h/m^2^)	10.54 ± 4.03	5.30 ± 1.65	6.04 ± 1.34	6.02 ± 1.35^a^
Vss (L/m^2^)	2.26 ± 0.81	1.87 ± 0.27	2.29 ± 0.20	1.98 ± 0.55^a^
MRT (h)	222.5 ± 52.6	371.2 ± 109.8	396.6 ± 123.3	341.0 ± 113.9^a^
Day 22 of cycle 1	*n* = 2	*n* = 3	*n* = 2	*n* = 6
C_max_ (μg/mL)	97.4	119.8 ± 11.3	238.8	535.5 ± 108.7
T_max_ (h)	4.56	0.99 ± 0.45	2.22	10.36 ± 10.11
AUC_(0-t)_ (μg•h/mL)	10134.5	14913.9 ± 1076.4	25555.3	72055.3 ± 22381.4
t_1/2_ (h)	–	249.7 ± 44.1	276.1	213.4 ± 85.1^b^

Mean ± SD

*C*_*max*_ maximum observed serum concentration; *T*_*max*_ time at the highest serum drug concentration was observed; *AUC* area under the serum concentration-time curve; *t*_*1/2*_ terminal elimination half-life; *CL* total body clearance; *V*_*ss*_ volume of distribution at steady-state; *MRT* mean residence time

^a^ *n* = 6

^b^ *n* = 3

### Clinical efficacy

**Fig. 3:**
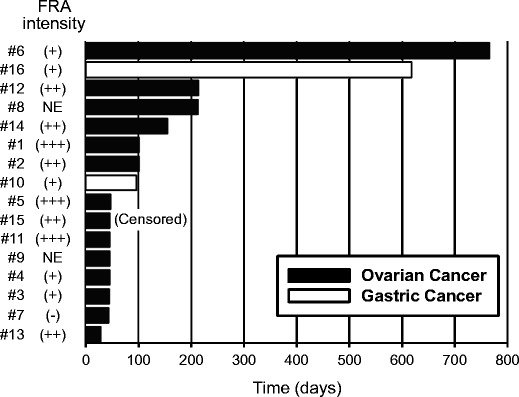
Progression-free survival of all patients

Of 16 total patients, one patient (#2) was excluded from analysis of tumor response because no measurable lesion was available. No major tumor shrinkage of partial or complete response was observed in the study. Eight of 15 evaluable patients (53.3 %) had stable disease, and 7 patients (46.7 %) had progressive disease as their best overall response. PFS for individual patients is shown in Fig. 3. Long-term disease stabilization was observed in one patient (#6) with clear cell OC treated with 100 mg/m^2^ for 772 days (25 months) and one patient (#16) with diffuse type GC treated with 400 mg/m^2^ for 628 days (20 months). No clear correlations between FRA expression level in archival tumor tissue and PFS were identified in this study; however, the sample size was limited.

## Discussion

Farletuzumab was generally safe and well tolerated in Japanese patients receiving up to 400 mg/m^2^ doses without encountering any DLTs, and the MTD was not reached at 400 mg/m^2^ in this phase I study. Adverse reactions evaluated by CTCAE were within grades 1 and 2 and were observed to be dose independent. Common adverse reactions were infusion related reaction, headache, nausea, and decreased appetite. These reactions were mild and well tolerated in all patients. Although 15 of 16 patients experienced infusion related reactions such as pyrexia, nausea, chills, and vomiting at first administration of farletuzumab, all of these reactions occurred within 24 h after the first administration and were managed without discontinuation of farletuzumab therapy. The safety profile of farletuzumab in Japanese patients was similar to that observed in a previous phase I study conducted in the US [10]. Positive HAHA was not detected in any patient in this study.

**Fig. 4:**
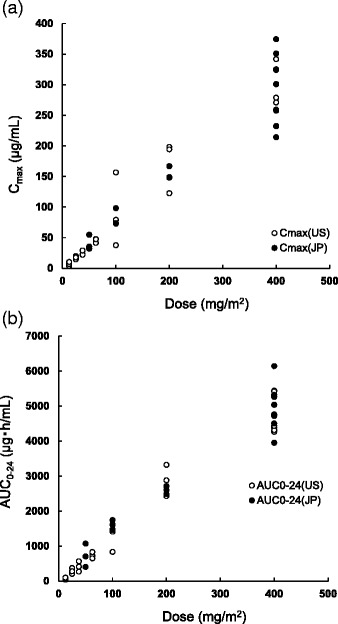
Relationship between dose and pharmacokinetic parameters of C_max_ (**a**) and AUC_(0-t)_ (**b**)

Pharmacokinetic parameters, including C_max_ and AUC_(0-t)_, increased dose dependently. A low clearance and a low distribution volume characterized the PK profile of farletuzumab, which is similar to the PK profiles reported by other anti-receptor antibodies [14]. When the relationship between the dose and the PK parameters of C_max_ and AUC_(0-t)_ on cycle 0 day 1 was compared with corresponding parameters observed in the previous US phase I study [10], the comparison revealed similar PK profiles of farletuzumab between Japanese and US patients (Fig. 4). Although the t_1/2_ value seems to be different between the Japanese and US studies, the difference can be attributed to the different sampling points (up to 504 and 168 h after administration for the Japanese and US study, respectively). The serum trough concentrations of farletuzumab after weekly repeated administration at the 50 mg/m^2^ dose level were more than 10 μg/mL, which is beyond the minimum concentration needed to induce ADCC and CDC in an in vitro pharmacology study [5].

Recently, it was reported in the results of a phase III trial that adding of farletuzumab to standard chemotherapy did not significantly improve PFS in platinum-sensitive recurrent OC [15]. The phase III trial was subsequently conducted using farletuzumab 1.25 and 2.5 mg/kg, equivalent to 50 and 100 mg/m^2^, respectively, according to the result of a US phase II trial [11]. In our study, we observed long-term stabilization in one patient with clear cell OC treated for 25 months at the dose of 100 mg/m^2^. Clear cell OC is recognized as a chemo-resistant subtype in OC. Actually, this clear cell OC patient was resistant to both paclitaxel/carboplatin and doxorubicin hydrochloride liposome injection. In addition, no DLTs were observed up to 400 mg/m^2^ of farletuzumab, suggesting that further clinical evaluation of farletuzumab at a higher dose may be warranted. The results of the phase III trial and subset analysis will be published in the near future, and a PK/pharmacodynamic analysis may provide some information about the recommended dose of farletuzumab in platinum sensitive recurrent OC.

In current study, there was no obvious relationship between the IHC intensity of FRA in archival tumor tissue at surgery and PFS in OC patients, who represent a relatively homogeneous patient background. FRA was only weakly expressed in the clear cell OC patient who demonstrated long PFS (Fig. 1b). FRA expression was also weak in the diffuse type GC patient who also had long-term disease stabilization (Fig. 1c). Although FRA expression in our study requires cautious interpretation because the sample size is small, our finding suggests that higher FRA expression may not correlate with better efficacy of farletuzumab. Cetuximab is an antibody for epidermal growth factor receptor (EGFR), but there is no clear relationship between expression level of EGFR and tumor shrinkage in phase II clinical trial for EGFR expressing colorectal cancer [16]. The clinical significance of FRA expression itself remains unclear in the treatment of farletuzumab. Continued biomarker research in both preclinical and clinical settings including candidate biomarkers such as an ADCC activity is still necessary for evaluation of farletuzumab in enriched patients.

This phase I trial is the first study where farletuzumab was administered to FRA-expressing non-OC patients, and we observed unexpected long-term disease stabilization in one patient with diffuse type GC. Recurrence after surgery was observed in the GC patient during treatment with S-1 (tegafur/gimeracil/oteracil) as an adjuvant setting, and salvage chemotherapy with two cycles of docetaxel failed. The PFS of the other GC patient was more than 3 months, which is also encouraging for treatment beyond second-line as compared with the results of second-line conventional chemotherapeutic agents [17, 18]. To best of our knowledge, this is the first report demonstrating the efficacy of farletuzumab in GC. GC is one of the most critical malignancies in East Asian countries including Japan. Both trastuzumab and ramucirumab are identified as an effective targeted agent in the treatment of GC [19, 20], however, further promising targeted drugs are required for prolonging survival. Although a phase III trial in patients with platinum-sensitive recurrent OC failed to show the efficacy of farletuzumab [15], our findings suggest that GC may be a promising target tumor type for farletuzumab therapy. Further preclinical and clinical evaluation of farletuzumab is warranted in the treatment of GC.

In conclusion, farletuzumab up to a dose of 400 mg/m^2^ was generally safe and well tolerated in Japanese patients with solid tumors. There were no DLTs and the MTD was not reached up to 400 mg/m^2^. The safety and PK profiles of farletuzumab were similar between Japanese and US patients. Long-term disease stabilization was observed in a subpopulation of clear cell OC and GC; both of them were resistant and progressive after standard chemotherapies.
